# Some thoughts on health insurance in the United States

**DOI:** 10.1002/acm2.12877

**Published:** 2020-04-13

**Authors:** Michael Mills

The AAPM is comprised of members with great diversity and with origins from many parts of the globe. There are some things about the culture in the United States that are no doubt unique and bewildering to those who have not grown up in the United States. One such peculiarity is the way hospital and other healthcare costs are paid. Many of us who are US natives will freely admit we can offer no logical reason why the situation is the way it is. Those of us who have taken the time to study the structure and history often come away no closer to understanding the swirl of money in healthcare than before. As someone who was involved in healthcare economic issues more than most in our organization, I will offer an introductory perspective to our diverse colleagues who have come to call the US their home.

My first real employment was in a hospital in 1969. I wanted to work in the kitchen, delivering food trays to patients. However, there were no openings, and instead I was offered a position as a radiology orderly, transporting patients from their hospital patient rooms to the x‐ray department and developing films in the dark room. (My introduction to medical physics was when two gentlemen came to visit one day per year to provide performance tests for our equipment.)

During the summer, I worked full time, and I noticed a pattern. Patients would check in on a Sunday and be scheduled for an array of morning procedures. One was performed on Monday, a second on Tuesday, and a third on Wednesday. Usually, these x‐ray tests were the intravenous pyelogram, the GI/gallbladder, and the barium enema. Always sequential and in this order. It did not take me long to think something was amiss with this arrangement. Could all of these patients have a medical disorder that would require all of these procedures? I also began to notice that the patients would return periodically for the same series of tests. When I asked the patients why they were here, they often replied: I am here for a rest. I began to wonder, who is paying for all of this? The short answer was hospital insurance, often Blue Cross and Medicare.

The origins of Blue Cross are found in Dallas, TX in 1929. Justin Ford Kimball, while he was vice president of Baylor University's healthcare facilities in Dallas, Texas, developed a plan to provide hospital coverage to teachers for up to 21 days for $6 per year. Before long, groups of hospitals were banding together to offer plans that were honored at all participating institutions, giving subscribers a choice of which hospital to use. This became the model for Blue Cross, which first operated in Sacramento, California, in 1932. An analogous type of plan and coverage was later extended to employees in lumber and mining camps of the Pacific Northwest to include coverage of physician costs; this was known as Blue Shield. (://en.wikipedia.org/wiki/Blue_Cross_Blue_Shield_Association); (://imprimis.hillsdale.edu/short-history-american-medical-insurance/). These are the earliest examples of employer‐paid health insurance.

Hospitals are very expensive to operate. The personnel, real estate, and operations are expensive; the equipment is not only very costly, but also becomes obsolete very quickly. Cash flow is always a problem because often patients require time to pay their bills. Hospital insurance provides a solution to this cash flow problem by providing reliable funds on an ongoing basis. No wonder this idea for hospital insurance proved popular with employees and spread very rapidly. It also had the benefit of generating steady and increasing demand for hospital services as they were already paid for. Additionally, the money does not return to the individual, as with most insurance; rather the funds are distributed directly to the hospital. While this obviously benefits the health institutions, it provides a perverse incentive. If patients do not care about the costs of their healthcare, they do not create a climate of competition between healthcare providers. There is no barrier from consumers of healthcare to keep costs in check. Physician care costs followed a similar economic model and patients were free to select mostly any physician regardless of the cost, unless the associated procedure was not covered or only partially covered.

In 1965, Congress proposed legislation to cover hospital and physician services, and what came of it were Medicare and Medicaid. President Lyndon B. Johnson signed it into law as part of his Great Society Legislation, capping 20‐yr of congressional debate. When the two programs were finally enacted, they were structured much like Blue Cross and Blue Shield, only with the government picking up much of the tab. In many states, the government actually hired Blue Cross/Blue Shield to administer the Medicare and Medicaid programs. The two new systems greatly increased the number of people who were eligible for advanced medical care, and the incomes of medical professionals soared, roughly doubling in the 1960s. Reimbursement for both hospitals and physicians were based on a fee‐for‐service procedure model. (://en.wikipedia.org/wiki/Blue_Cross_Blue_Shield_Association); (://imprimis.hillsdale.edu/short-history-american-medical-insurance/). This history explains much of my experience in 1969; some of the structure is still with us, in a modified form.

This structure sets the stage for the following dynamic: The government wants to pay less for hospital and physician services, so it does where it can. Hospital and physician groups band together to become large enough to negotiate directly with the carriers. The results of these negotiations are not public information. The financial well‐being of healthcare institutions becomes based more on the size of the population it serves than the cost and quality of its services. In days past, there was a robust relationship between community leaders and the hospitals, which were under the control of local boards. Religious‐based hospitals received support from churches and other community organizations. Sadly, this local interest and enthusiasm no longer exists for many hospitals and local boards often have little power over important decisions. Therefore, there is also a necessary loss of local community control of hospitals and associated healthcare delivery.

There is much additional complexity to master: the growth of HMOs and PPOs, the motivation behind capitation for services, the expansion of the power of state governments over healthcare and hospitals, and the significance of malpractice, and other litigation in the healthcare arena.

What should happen next? It is necessary to get consumers to care again about their personal healthcare costs. Some mechanism should be found to make the entire system transparent as to costs and charges. Consumers must bear responsibility for healthcare purchases, which means that they should be responsible for some portion of the costs, and it should be proportionate to the newly transparent charge and fee schedules in order to promote competition among providers.

What will all this mean for medical physicist salaries and program support? My magic 8‐ball says the answer is unclear; try again later.

